# Electrophysiological Markers of Proactive and Reactive Cognitive Processing in Post-Traumatic Stress Disorder

**DOI:** 10.3390/brainsci16080810

**Published:** 2026-07-30

**Authors:** Biancamaria Di Bello, Raffaele Costanzo, Natalie Ferrulli, Margherita Filosa, Maria Gabriela Bevacqua, Francesca Strappini, Valentina Sulpizio, Francesco Di Russo, Sabrina Pitzalis

**Affiliations:** 1 Department of Movement, Human and Health Sciences, University of Rome “Foro Italico”, 00135 Rome, Italysabrina.pitzalis@uniroma4.it (S.P.); 2 LIFE Institute for Research and Health Care Santa Lucia IRCCS, 00179 Rome, Italy; 3 Studio Psyche Neuroscienze, 00192 Rome, Italy; 4 Department of Humanities, Education and Social Sciences, University of Molise, 86100 Campobasso, Italy

**Keywords:** EEG, ERP, PTSD, anticipation, decision-making

## Abstract

**Highlights:**

**What are the main findings?**

- PTSD patients show a broad suppression of anticipatory ERP, indicating a generalized deficit in motor, cognitive, and sensory preparation before task execution.
- Reactive processing in PTSD is selectively impaired at higher cognitive levels: early perceptual components are intact, while the P3, reflecting decision-making and task closure, is reduced.

**What are the implications of the main findings?**

- Reduced anticipatory brain activity in PTSD may be consistent with altered functioning of prefrontal, cingulate, and visual associative networks, supporting these circuits as candidate neural substrates of proactive cognitive control deficits.
- Pre-stimulus ERP components represent promising electrophysiological biomarkers for clinical assessment of PTSD, capturing dysfunction that is not detectable through behavioural accuracy measures alone.

**Abstract:**

**Background:** Post-Traumatic Stress Disorder (PTSD) is associated with well-documented alterations in reactive brain processing of stimuli or events, whereas little is known about proactive/anticipatory neural dynamics preceding and preparing for task execution. This study aimed to investigate both proactive and reactive brain control of PTSD patients during a cognitive task, using the event-related potential (ERP) method. **Methods:** Twenty-one PTSD patients were matched to thirty healthy controls. Proactive ERP components, such as the Bereitschaftspotential (BP), prefrontal Negativity (pN), and visual Negativity (vN), were analysed to assess motor, cognitive, and sensory preparation, respectively. Reactive components such as the P1, N1, and P3 were also analysed to evaluate perceptual, attentional, and decisional processes, respectively. **Results:** Results revealed in PTSD patients a marked reduction in the amplitudes of all anticipatory components, suggesting a generalized impairment of top-down control in task preparation. Analysis of post-stimulus reactive processing revealed no group differences in early components associated with perceptual and attentional processing (P1, N1), but a reduction of the P3 in the PTSD group, indicating impaired higher-order cognitive processing related to decision-making and task closure. Behaviourally, patients exhibited longer and more variable response times, although accuracy remained unaffected, consistent with a speed-accuracy trade-off. **Conclusions:** To our knowledge, this is the first ERP study to examine anticipatory brain activity in PTSD through the BP, pN, and vN components. These results provide evidence of widespread proactive processing deficits in PTSD, consistent with previous reports of altered functioning within prefrontal, cingulate, and visual association networks. Moreover, anticipatory ERP components may represent promising neurophysiological markers for the assessment of cognitive dysfunction in PTSD.

## 1. Introduction

Post-traumatic stress disorder (PTSD) is a mental health condition whose onset is directly or indirectly linked to the experience of traumatic, or, in some cases, highly stressful events, which may result in clinically significant psychological trauma in a substantial proportion of exposed individuals. Trauma can be defined as psychological harm resulting from an event that overwhelms an individual’s ability to cope with or integrate the emotions and experiences associated with it [1]. However, the critical factor is how the individual perceives, experiences, and internalizes the event. This suggests that a potentially traumatic event may be subjectively perceived as non-traumatic and therefore may not necessarily lead to the onset of the disorder [2].

From a neurobiological perspective, PTSD is thought to be facilitated by dysfunctions in neurotransmitter systems, which lead to altered stress responses [3]. The traditional neurobiological model of PTSD [4] attributes the disorder’s origin to alterations in the cortico-subcortical network, which includes the prefrontal cortex (PFC), parietal regions, and limbic structures, such as the amygdala and hippocampus [5,6,7,8]. Specifically, amygdala hyperactivity [9,10] is thought to occur in conjunction with inadequate regulation by the PFC, which may be due to either functional impairments [4,6] or structural reduction [11,12]. Additionally, a reduction in the hippocampal volume is associated with the memory deficits observed in individuals with PTSD [13,14,15,16]. This alteration, when combined with structural [17] and functional [18,19] reductions in the cingulate cortex, impairs the ability to contextualize past experiences in a coherent framework [20,21]. This dysfunction compromises the categorization of experiences as either threatening or non-threatening and contributes to the hallmark symptoms of avoidance and re-experiencing. Furthermore, dysfunctions in the visual association cortex have also been linked with the disorder [18,19].

The neural correlates of PTSD have been extensively studied using event-related potential (ERP) methodologies, particularly in experimental paradigms involving sensorimotor cognitive discrimination tasks with affective or non-affective stimuli. These studies have consistently identified systematic alterations in various ERP components following stimulus onset in patients with PTSD. Specifically, they found enhanced early processing of affective stimuli and impaired late processing of non-affective stimuli [22]. Research utilizing non-affective visual stimuli has shown no dysfunction in early stimulus processing, as indexed by the P1 and N1 components. However, reduced amplitudes of the P3 component were found in response to both target and non-target stimuli, as well as diminished P3a amplitudes in response to unexpected novel stimuli [23]. The P3 component is associated with post-perceptual attentional and decisional processes in the posterior parietal cortex and limbic regions, including the hippocampus. In PTSD, the reduction in P3 amplitude has been linked to difficulties in distinguishing stimulus relevance, likely resulting from disrupted concentration and working memory deficits, which may be attributed to noradrenergic and hippocampal dysfunctions [24,25,26,27,28,29,30]. Furthermore, Felmingham et al. [25] found a negative correlation between the intensity of numbness symptoms and P3 amplitude. These findings are in line with models positing a relationship between dysregulated arousal and attentional processes in PTSD. Thus, they support the hypothesis that P3 abnormalities reflect a reduction in cognitive processing, which impairs stimulus discrimination and the inability to allocate attentional resources effectively. Recent studies have further supported the relevance of electrophysiological measures as objective markers of PTSD, highlighting the potential of EEG and ERP indices for characterizing symptom dimensions and developing precision-medicine approaches to diagnosis and treatment monitoring [31]. In parallel, large-scale reviews have emphasized the growing role of EEG- and ERP-based biomarkers, including P300 measures, in the objective assessment of PTSD and related cognitive dysfunctions [32].

The studies reviewed above primarily investigated reactive brain processing, namely neural responses elicited after stimulus presentation, and consistently reported alterations in the P3 component, which has been associated with higher-order cognitive processing. However, no studies have yet explored proactive brain processing in PTSD—defined as the brain’s preparation and anticipation of upcoming events or tasks, based on the active maintenance of goals/context to guide future processing [33]. This represents a significant gap in the PTSD literature, as anxiety is inherently anticipatory, and reduced predictive control has been documented in individuals with PTSD [34]. Thus, investigating anticipatory brain processing in PTSD is crucial for identifying neural correlates of predictive control dysfunction, potentially linked to the hypoactivity in the PFC and cingulate areas [4,6,18,19].

To examine proactive brain processing using ERP techniques, three components may be particularly relevant. The most well-known of these is the Bereitschaftspotential (BP), or readiness potential. The BP is a slow negative wave associated with motor preparation and the planning of voluntary movements [35]. It reflects the progressive excitation of the supplementary motor area (SMA) and cingulate motor area (CMA) in anticipation of upcoming movements. Larger BP amplitudes are associated with faster response times in sensory-motor tasks [36]. Another anticipatory component is prefrontal negativity (pN), which is localized in prefrontal areas and related to cognitive preparation, particularly top-down inhibitory control [37]. This component is localized in the PFC [38] and is reduced under conditions of increased anxiety [39], a core symptom of PTSD. The pN differs from the contingent negative variation (CNV), which reflects temporal expectancy elicited by cues predicting an upcoming target. Instead, the pN emerges in complex tasks independently of cues [40,41] and represents different brain functions [36,42]. Finally, the visual negativity (vN) is an anticipatory component linked to sensory readiness [43]. It reflects the generation of a low-level mental representation of the upcoming stimulus to enhance perception [44,45]. Larger vN amplitudes have been associated with faster response time [36].

Based on this gap in the literature, the present study addressed three main research questions: (1) Are proactive ERP components associated with motor, cognitive, and sensory preparation (BP, pN, and vN) altered in PTSD? (2) Do PTSD patients show abnormalities in reactive processing limited to late cognitive stages (P3), while preserving early perceptual and attentional processing (P1 and N1)? (3) Are neurophysiological alterations associated with behavioural impairments such as slower and more variable response times?

The present study aimed to investigate both proactive and reactive cognitive processing in PTSD using event-related potentials during a visuomotor discrimination task with non-affective stimuli. By examining both anticipatory and post-stimulus ERP components within the same experimental paradigm, we sought to characterize whether cognitive dysfunction in PTSD primarily affects proactive control, reactive processing, or both.

Of particular significance, the present study examined, to our knowledge for the first time, the neural mechanisms underlying motor, cognitive, and visual anticipatory processing, indexed by the BP, pN, and vN components, respectively, in a PTSD population. We formulated the following hypotheses. First, PTSD patients would show reduced amplitudes of the anticipatory BP, pN, and vN components, reflecting impaired motor, cognitive, and sensory preparation. Second, we expected no significant group differences in the early reactive components (P1 and N1), consistent with previous ERP findings obtained with non-affective stimuli. Third, we expected a reduced P3 amplitude in PTSD patients, indicating impaired higher-order cognitive processing. Finally, we predicted slower and more variable behavioural responses in PTSD, accompanied by preserved or only minimally affected accuracy.

Slowing of response times may correlate with the suppression of the BP and vN components and could be considered an index of cognitive inefficiency. The slowing is consistent with deficits in sustained attention, working memory, and inhibitory control, which typically manifest as impaired interference control in the presence of emotional or trauma-related stimuli, but result in delayed evaluation when processing non-emotional, trauma-unrelated stimuli [46,47]. These difficulties in filtering out generic interference [46,48] result in less stable executive control. In light of previous research suggesting that increased response time variability serves as a marker of executive dysfunction in PTSD [49], we expect to observe an increase in the intraindividual coefficient of variation. Furthermore, we hypothesize a reduction in response accuracy in PTSD patients due to the suppression of the pN component. However, following the speed/accuracy trade-off principle, slower response times may compensate for this accuracy dysfunction, as seen in older healthy people [37].

## 2. Methods

### 2.1. Participants

The sample size was determined through a power analysis conducted using G*Power 3.1.9.7 software [50]. According to the meta-analytic review on PTSD ERP studies of Karl et al. [23], because no previous studies have examined BP, pN, or vN alterations in PTSD, sample-size estimation was necessarily based on the largest effect reported for the P3 component. We set the effect size to d = 0.81, which was the reported largest effect size for *t*-tests between P3b amplitude of PTSD patients and controls. The alpha level was set to 0.05 and the power to 0.80. These parameters resulted in a minimum required sample size of 50 participants. Accordingly, 24 PTSD patients and 30 healthy controls were recruited. Three PTSD participants were lost because they did not perform the EEG assessment. Therefore, the final sample consisted of 21 PTSD patients (mean age = 35.1 ± 17.1 years, 61% females) and 30 healthy controls (mean age = 37.6 ± 10.9 years, 50% females). Preliminary analyses revealed no significant differences between the two groups in terms of numerosity, sex (X2(1) < 1), and age (t(49) < 1).

The diagnosis of PTSD was made based on the criteria outlined in the Diagnostic and Statistical Manual of Mental Disorders, 5th Edition, Text Revision (DSM-5-TR). All PTSD patients had a history of at least one traumatic event, including physical or emotional abuse, childhood attachment trauma, physical or emotional neglect, abandonment, witnessing domestic violence, severe accidents, bereavement, relationship loss, or living with a pathological dependent individual, such as an alcoholic, drug addict, or pathological gambler. Other inclusion criteria for PTSD patients were as follows: (1) legal capacity to consent to treatment; (2) age between 18 and 65 years. Exclusion criteria included: (1) a history of schizophrenia, psychotic symptoms, bipolar disorder, dementia or head trauma, or severe personality disorder; (2) any substance abuse or addictive disorder (excluding nicotine) within the past six months; (3) severe visual impairments. All PTSD participants met criteria for chronic PTSD, with symptoms persisting for more than six months before assessment.

The control group comprised individuals matched to the experimental group by age and sex. The same exclusion criteria were applied, with the following inclusion criteria: (1) absence of traumatic events or PTSD symptoms, as verified by clinical interview; (2) age between 18 and 65 years; (3) the legal mental capacity to provide informed consent for participation; and (4) normal or corrected vision. Notably, control participants were only assessed through the clinical interview to confirm the absence of psychiatric disorders and were not administered self-report questionnaires.

**Figure 1:**
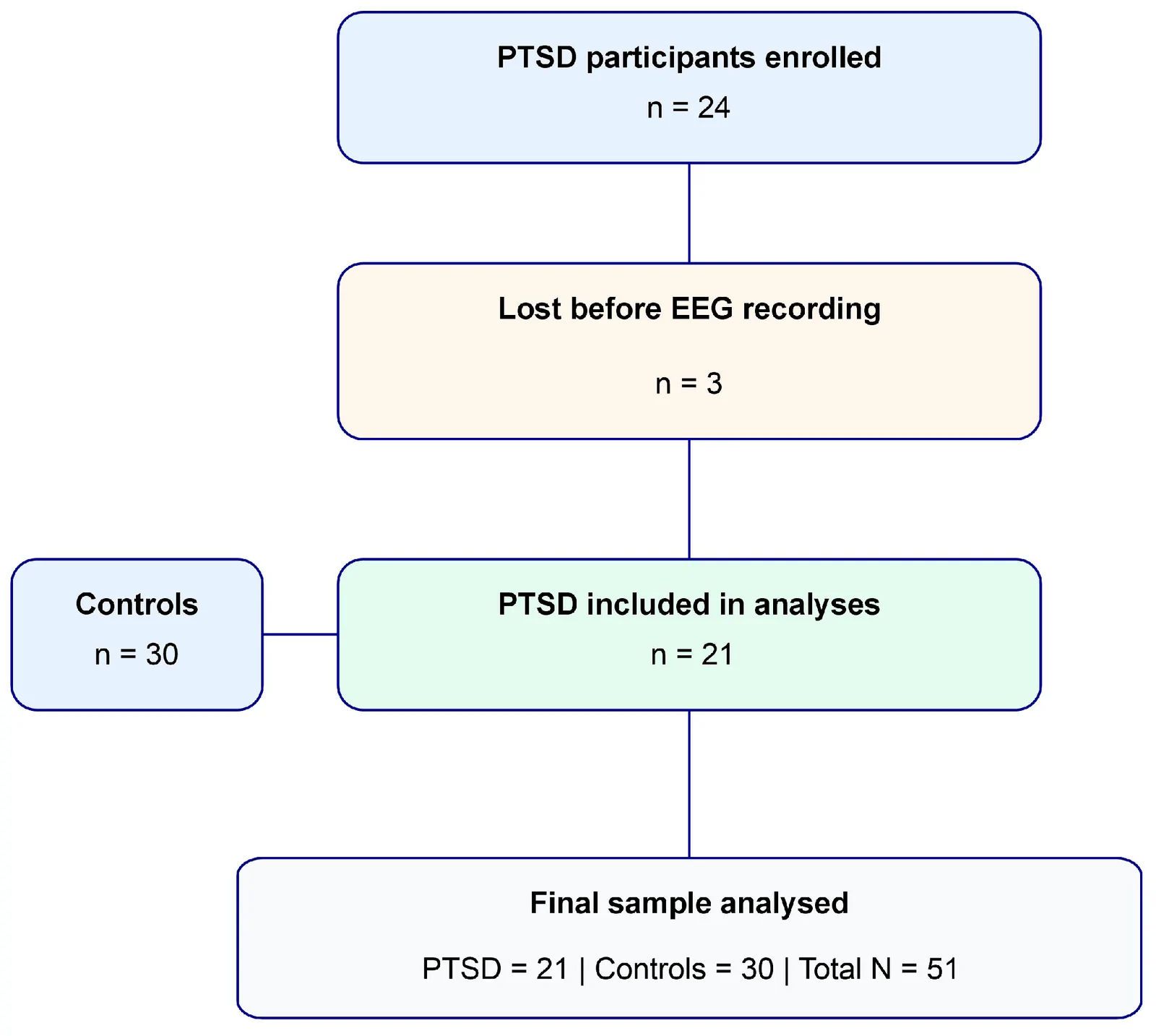
Participant flow diagram. Twenty-four individuals with PTSD were initially enrolled. Three participants were lost because they did not perform the EEG assessment. The final sample included 21 PTSD patients and 30 healthy controls.

A flow diagram of participant recruitment and retention is provided in Figure 1.

PTSD participants were recruited between January 2025 and March 2026 from individuals seeking psychological treatment at Studio Psyche Neuroscienze (Rome, Italy). Control participants were recruited from students and staff at the University of Rome “Foro Italico.” All participants voluntarily consented to take part in the study and were naïve to the experimental objectives. The study adhered to the ethical standard set forth by the World Medical Association (Declaration of Helsinki) and received approval from the research ethics committee of the University of Rome “Foro Italico,” obtained with protocol number CAR 74/2023.

### 2.2. Procedure

Diagnostic evaluation was performed by senior clinicians with expertise in trauma-related disorders and naïve to the experimental objectives. In addition to confirming DSM-5-TR PTSD diagnosis, the assessment excluded schizophrenia spectrum disorders, bipolar disorder, dementia, severe personality disorders, and current substance-use disorders. Self-report questionnaires were further used to assess the presence of PTSD symptoms. The following standardized questionnaires were administered: the State-Trait Anxiety Inventory [51]; the Beck Depression Inventory-II [52]; the Impact of Event Scale-Revised [53]; the Dissociative Experiences Scale [54]; and the Adverse Childhood Experience questionnaire [55]. The outcomes of these questionnaires are reported in the Clinical Outcomes Result section to characterize the present PTSD group, but no analyses were made on them. None of the PTSD patients were taking anxiolytic, antidepressant, antipsychotic, or other medications potentially affecting cognitive or electrophysiological measures. EEG data were collected before the initiation of psychotherapeutic treatment. In addition, PTSD patients did not report neurological or relevant physical diseases that could interfere with cognitive performance or EEG recordings. Participants with a history of head trauma, severe visual impairment, neurological conditions, or other medical conditions potentially affecting the experimental procedure were not included.

**Figure 2:**
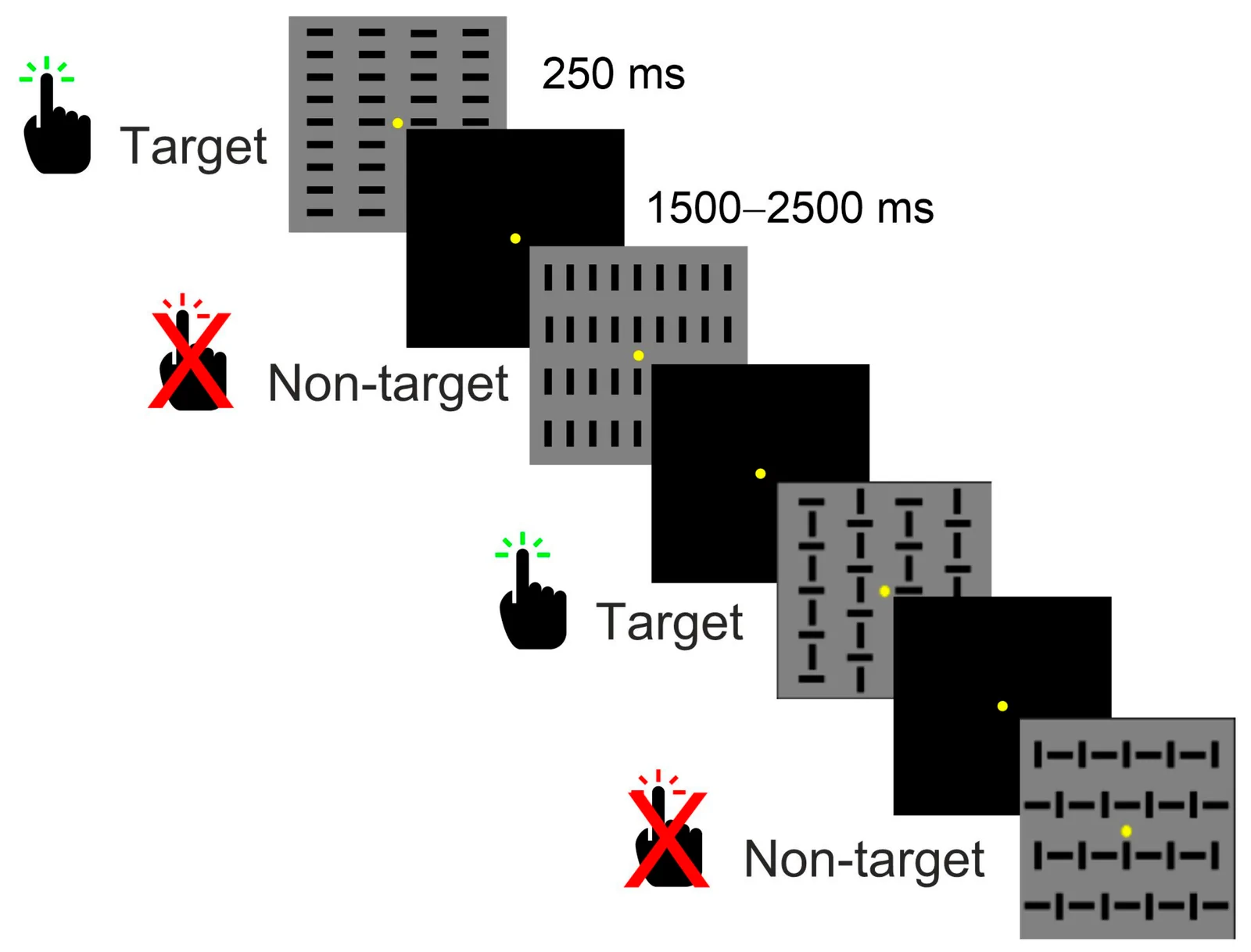
Representation of the cognitive task, with features and timing of the stimuli.

To assess cognitive functioning, participants completed a discriminative response task (DRT), specifically an equiprobable Go/No-go paradigm, during EEG recording. We used this task since it generates strong stimulus-locked anticipatory ERPs such as the pN, BP, and vN, which we predicted to be modulated in PTSD patients. Additionally, this task is useful to isolate intrinsic anticipatory/preparatory processes from expectancy-driven effects that arise when stimulus probabilities or cues bias the participant toward one response. For this task, participants were seated in a dimly lit, sound-attenuated room after the EEG cap was fitted to the scalp. They were positioned in front of a 32-inch monitor at a distance of 114 cm with a black screen displaying a central yellow fixation point (0.15° × 0.15°) that remained in place for the whole run. The response was made by pressing a button, which participants held in their right hand, resting on their right leg. Before the experiment, participants received explicit instructions to fixate on the designated point and to suppress blinking and other ocular movements as much as possible during task performance. The task required participants to respond (i.e., press the key) to target stimuli and refrain from responding to non-target stimuli. Two target stimuli and two non-target stimuli appeared in rapid succession, in random order with equal probability (*p* = 0.25). Each stimulus appeared for 250 ms, with the interstimulus interval ranging from 1500 to 2500 ms to mitigate potential learning effects. The stimuli were 4°x4° grey squares respectively containing vertical, horizontal lines or both orientations. The non-target stimuli resembled the target stimuli but were rotated by 90° relative to the target stimuli (Figure 2). Specifically, target stimuli contained horizontal lines or vertical lines overlaid with horizontal lines, while non-target stimuli contained vertical lines or horizontal lines followed by vertical lines. The use of two targets and two non-targets was functional to keep the task difficulty high and therefore to obtain large and reliable pN and BP components [56]. Participants performed 10 runs of this task, resulting in a total of 800 trials (400 target stimuli and 400 non-target stimuli). Participants were instructed to respond as quickly as possible while maintaining accuracy. Midway through the session, participants were allowed to take a short break. The total duration of the EEG recording was between 35 and 40 min.

### 2.3. Data Recording and Analysis

The EEG signal was recorded using the BrainAmpTM system (BrainProducts GmbH, Munich, Germany) with 64 scalp electrodes mounted according to the 10-10 International system. All electrodes were initially referenced to the left mastoid and then re-referenced to both mastoids. Horizontal and vertical electrooculogram (EOG) data were also recorded with bipolar montage, with electrodes placed at the right external canthus and below the left eye, respectively. Electrode impedances were maintained below 5 KΩ. The EEG was digitized at 250 Hz, amplified with a band-pass (0.01–80 Hz including a 50 Hz notch filter), and stored for offline analysis using BrainProducts’ Analyzer 2.3 software. Eye movement artifacts were corrected using the Analyzer’s built-in regression-based procedure, which implements the Gratton, Coles, and Donchin [57] method by removing EOG-related activity from the other channels. Artefact rejection was then performed to discard epochs contaminated by signals exceeding the amplitude threshold of ±60 μV, ensuring the removal of potential non-ocular artefacts. On average, 5% of trials were rejected, leaving 760 and 380 trials for the pre- and post-stimulus ERP, respectively. No differences in terms of blink number and rejection rate were found between groups (t < 1).

For pre-stimulus ERP, the EEG was segmented into 1300 ms epochs, synchronized with stimulus onset, spanning from 1100 ms before to 200 ms after the stimulus. The baseline was set from −1100 to −900 ms. For the post-stimulus ERP, the EEG was segmented into 1000 ms epochs, synchronized with stimulus onset, extending from 100 ms before to 900 ms after the stimulus. The baseline was defined from −100 to 0 ms. Artefact-free trials were averaged into three sets of ERPs: (1) the pre-stimulus ERP, including all trials; (2) the post-stimulus target trials; and (3) the post-stimulus non-target trials. Correct trials only were included.

To identify scalp sites and the time intervals for subsequent statistical analyses, a spatiotemporal cluster permutation analysis based on the Wilcoxon test was employed [58]. The cluster analysis was performed on two localizers: one for pre-stimulus data, averaging the ERP of the two groups, and another for the post-stimulus data, averaging the ERP of the two groups and two trial types (target and non-target). The two localizers were compared to zero using the following parameters: time range from −900 to 0 ms for pre-stimulus data and from 0 to 900 ms for post-stimulus data; 10,000 randomizations; critical alpha 0.01; minimum spatial cluster size of 3 channels; cluster alpha 0.01; and minimum cluster duration of 10 samples (40 ms) for pre-stimulus data and 3 samples (12 ms) for post-stimulus data.

For pre-stimulus data, the analysis revealed a single temporal cluster from −708 to 0 ms, with three spatial clusters: one prefrontal cluster (Fp1, Fpz, Fp2, AF7, AF3, AFz, AF4, AF8 electrodes), associated with the pN component; one central cluster (C1, Cz, C2, CP1, CPz, CP2 electrodes) associated with the BP component; one occipital cluster (PO1, POz, PO2, O1, Oz, O2 electrodes), associated with the vN component. For post-stimulus data, three temporal clusters were identified: from −116 to 140 ms, from 184 to 212, and from 472 to 556 ms, which were associated with the P1, N1, and P3 components, respectively. In the first two intervals, the same parieto-occipital spatial cluster was detected, including the PO7, PO3, O1, O2, PO4, and PO8 electrodes. In the P3 interval, a parietal spatial cluster was identified, including the CP1, CPz, CP2, P1, Pz, and P2 electrodes. The mean amplitudes of these spatial and temporal clusters were calculated for statistical analyses. Voltage maps from a top-flat view were used to visualize the scalp topographies of the studied components in the identified intervals. In addition, current source density (CSD) maps were also used since the Laplacian transformation of the voltage scalp distribution removes the influence of the reference electrode, improving scalp spatial resolution and facilitating visualization of local current gradients. This is particularly useful in the pre-stimulus interval, where the voltage maps may hide the activity of components overlapping over time. The spherical spline interpolation method was used to display the maps, setting the order of splines to 4 and a maximum degree of Legendre polynomials to 10. Since applying CSD requires that all scalp electrodes be free of artifacts, contaminated channels were interpolated at the individual level. The average number of interpolated channels per participant was 2.5 ± 3.3, ranging from 0 to 6. EEG analyses were conducted on raw voltage data; CSD transformation was used for mapping purposes only.

Behavioural data were obtained by measuring the mean response time (RT) for target trials and its variability (RT standard deviation divided by the RT and transformed into percentage of RT variability). Accuracy was assessed by calculating the error rate, which was derived from the sum of the missed responses (i.e., omitted responses to target stimuli) and false alarms (i.e., responses to non-target stimuli).

### 2.4. Statistical Analysis 

A *t*-test for independent samples was employed to compare the RT, the RT variability, accuracy, and the amplitude of the pre-stimulus ERP components (pN, BP, and vN) between the two groups. For the post-stimulus ERP components (P1, N1, and P3) amplitude, a 2 × 2 mixed ANOVA analysis of variance design was performed with Group as the between-subjects factor and Trial (target vs. non-target) as the within-subjects factor. Effect sizes were reported in terms of Cohen’s *d* for *t*-tests and partial eta squared (η_p_^2^) for ANOVAs. The overall alpha level was set at 0.05. The statistical analyses were performed using Statsoft Statistica version 12 (StatSoft, Inc., Tulsa, OH, USA).

## 3. Results

### 3.1. Clinical Outcomes

All participants in the PTSD group met the DSM-5-TR PTSD criteria. Regarding the clinical assessments, it was observed that the patients exhibited elevated levels of PTSD symptoms, as indicated by the IES-R scores (mean = 42.4, SD = 18.0). Specifically, the mean score for the Avoidance subscale (IES-Evit) was 1.7 (SD = 0.8), the mean score of the Intrusiveness subscale (IES-Intro) was 2.0 (SD = 1.0), and for the Hyperactivation component (IES-Iper), the mean score was 2.2 (SD = 1.0).

For state anxiety (STAI-Y), the mean score obtained was 49.2 (SD = 14.3), while for trait anxiety (STAI-X), the mean score was 53.7 (SD = 11.9), suggesting a probable clinical level of anxiety. The assessment of dissociative experiences using the Dissociative Experiences Scale (DES-2) showed a mean of 18.5 (SD = 13.7), suggesting a moderate level of dissociative experiences among the participants.

Concerning adverse childhood experiences, the mean score on the Adverse Childhood Experiences (ACE) was 2.7 (SD = 2.2), indicating a relatively low prevalence of adverse experiences in the sample. The Beck Depression Inventory (BDI) yielded a mean of 17.7 (SD = 11.9), reflecting a moderate intensity of depressive symptoms in the patient group.

### 3.2. Behavioural Data

**Figure 3:**
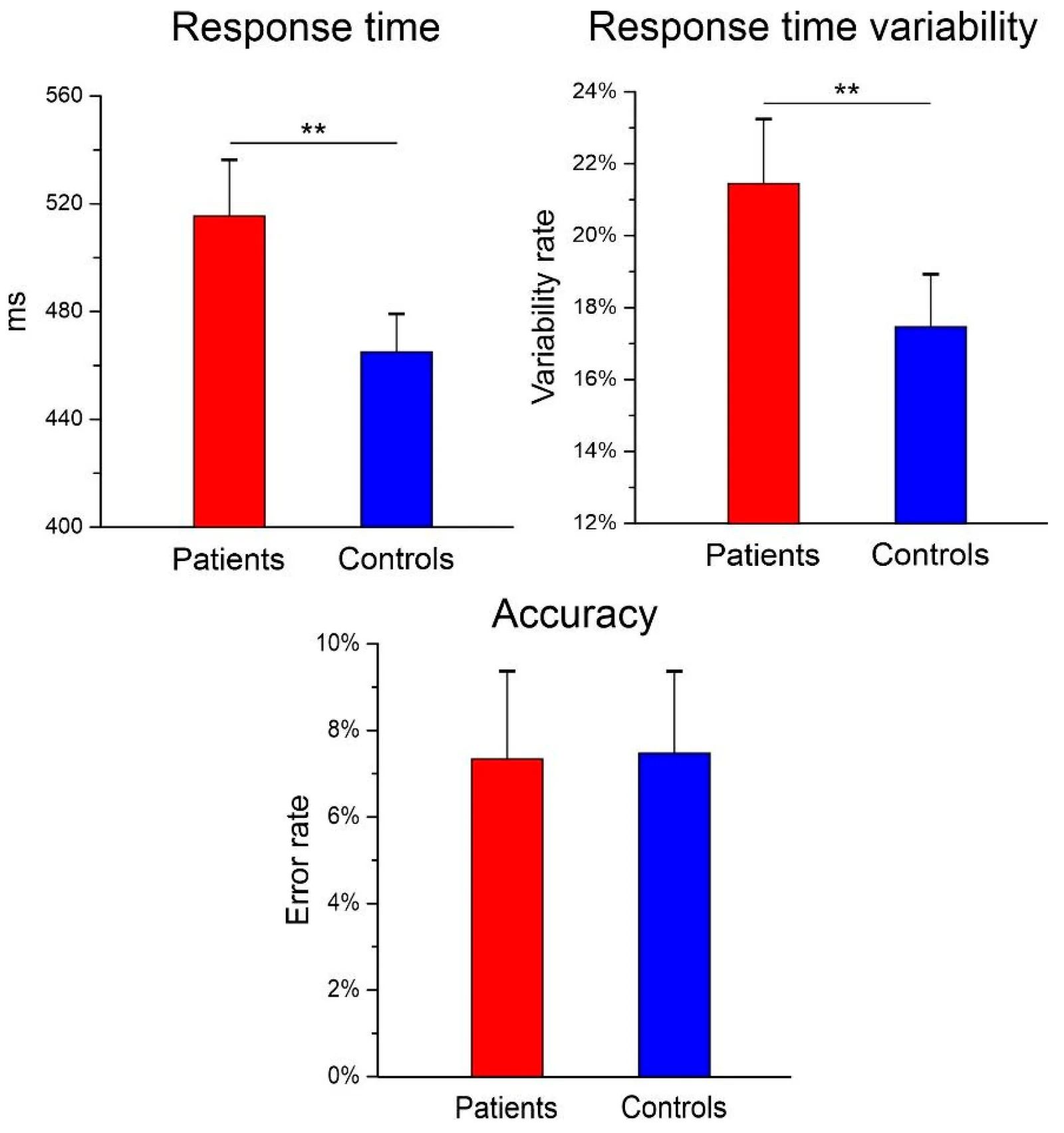
Behavioural data of the two groups showing the mean response time, variability, and accuracy. The vertical lines represent the 95% confidence interval. Significant group differences are indicated (** *p* < 0.01).

Independent-samples *t*-tests revealed significant group differences in both response time (RT) and response-time variability (ICV). PTSD patients showed slower responses (M = 515 ms, SD = 52) than healthy controls (M = 458 ms, SD = 40), t_(49)_ = 4.19, *p* < 0.001, d = 1.25. 95% CI of the mean difference (29,84) ms. Furthermore, patients exhibited greater intraindividual response-time variability (M = 21.3%, SD = 6.5) compared with controls (M = 17.5%, SD = 4.0), t_(49)_ = 2.39, *p* = 0.023, d = 0.74, 95% CI (0.55%, 7.03%). In contrast, no significant group difference emerged for response accuracy (t_(49)_ = 0.03, *p* = 0.980, d = 0.01). The mean error rate was 7.5% (SD = 6.9) in the PTSD group and 7.4% (SD = 5.2) in the control group, with a mean difference and 95% CI of (−3.55%, 3.64%). Figure 3 provides a graphical summary of the behavioural results.

### 3.3. ERP Data

**Figure 4:**
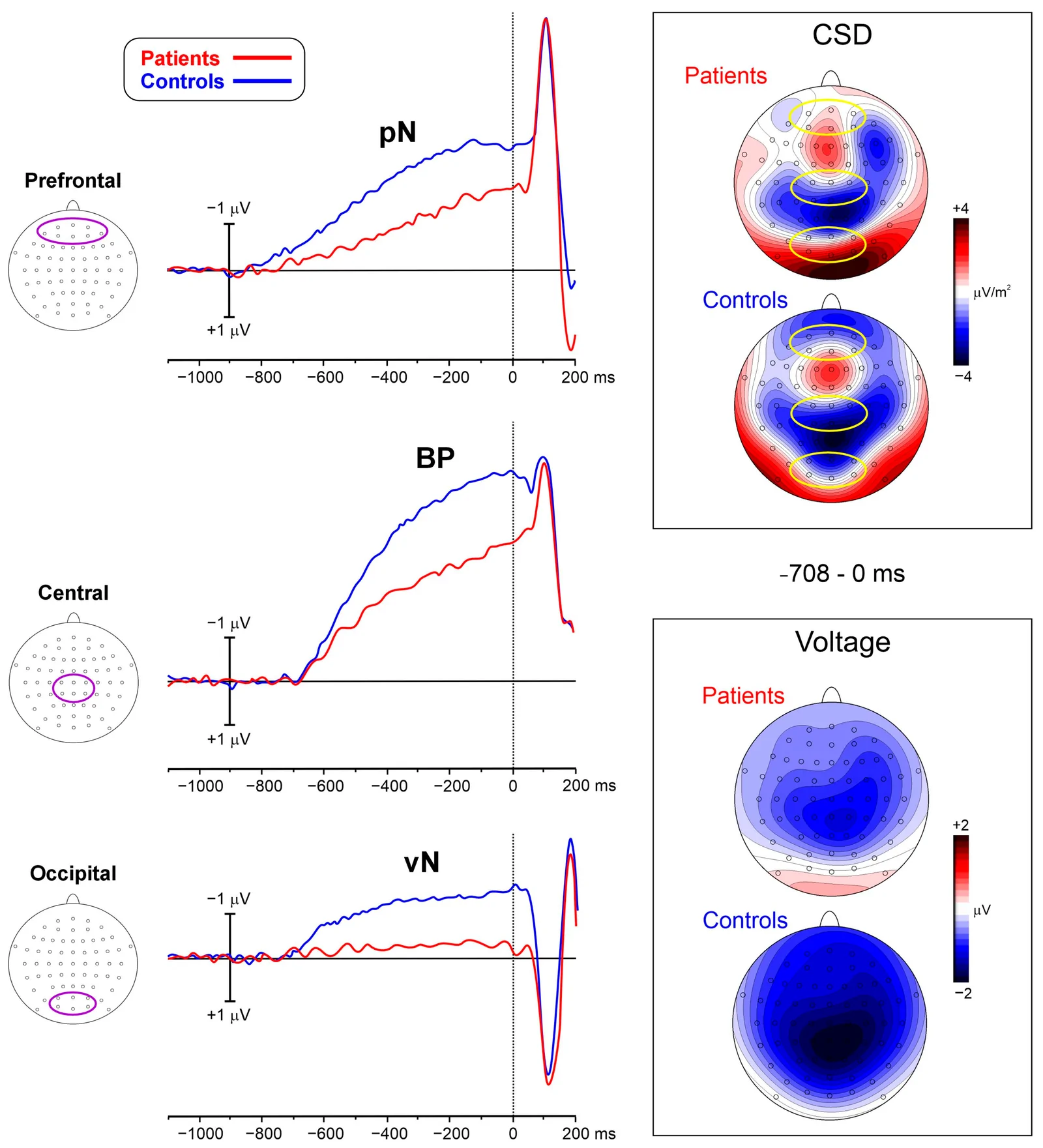
**Left**: Pre-stimulus ERP waveforms of the two groups. The circles within the head figures indicate the electrodes included in the waveforms. **Right**: current source density (CSD) and voltage scalp topography (top-flat view) in the −708/0 ms interval. The circles within the CSD maps indicate the possible localization of the three pre-stimulus ERP components (from top to bottom: pN, BP, and vN).

**Figure 5:**
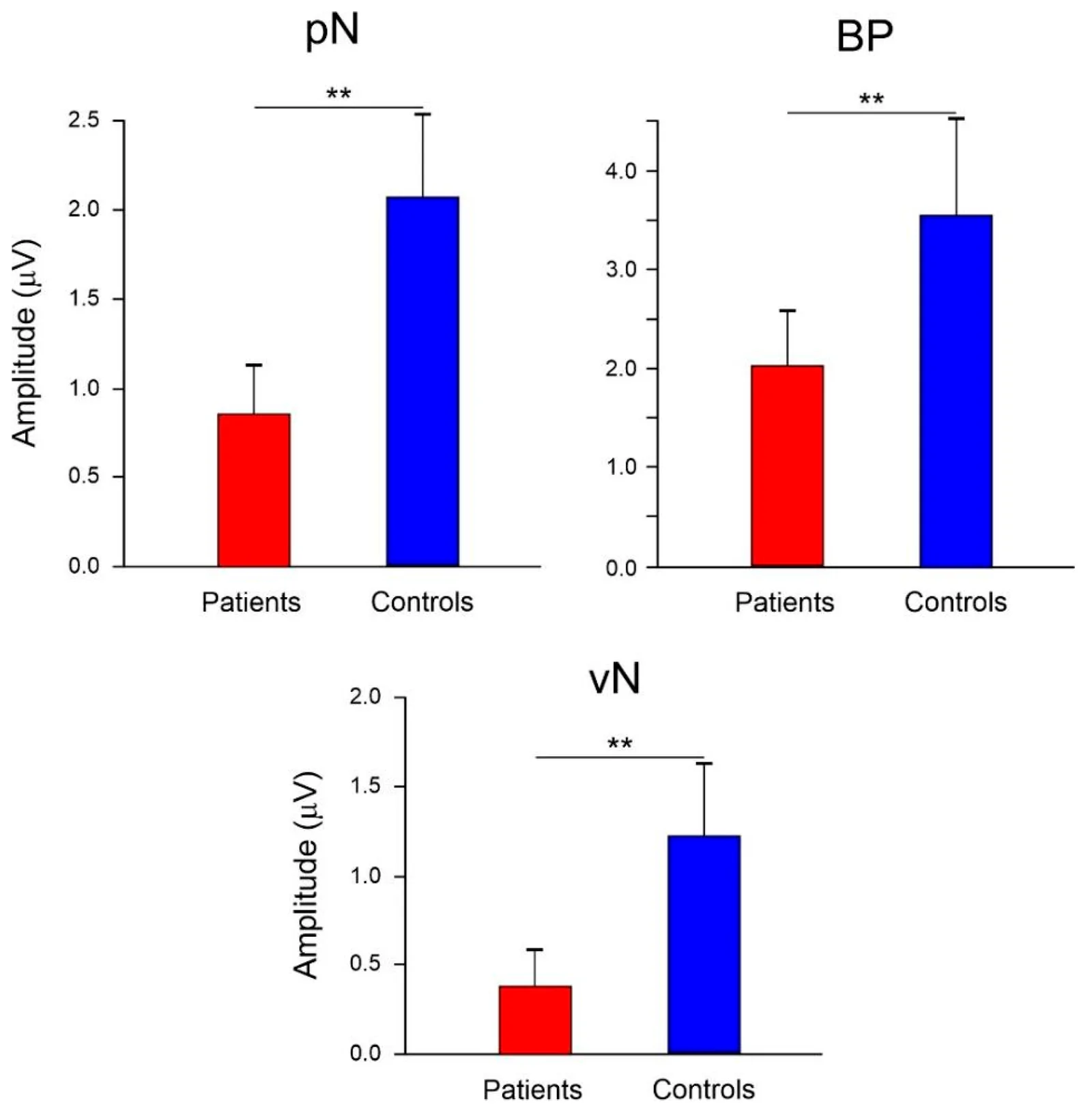
Amplitudes of the pre-stimulus components and their variability expressed as the 95% confidence interval. Significant differences are also indicated (** *p* < 0.01).

The left side of Figure 4 displays the pre-stimulus ERP waveforms for the two groups across the three clusters of electrodes representing the pN, BP, and vN components. The right side of Figure 4 shows the voltage and CSD pre-stimulus scalp topography in the −708/0 ms intervals. In the CSD maps, ovals indicate the three potential current sources-prefrontal, central, and occipital current—as depicted by the maps. *t*-tests carried out on the mean amplitude in the −708 to 0 ms interval revealed significant differences for all components (t_(49)_ > 6.5, *p* < 0.01), indicating lower amplitudes in the patient group. Specifically, the pN was −0.85 μV (SD = 0.51) and −2.08 μV (SD = 0.89) for the patient and control group, respectively (d = 1.69). The BP amplitude for the patient group was −1.98 μV (SD = 1.33), compared to −3.49 μV (SD = 1.47) in the control group (d = 1.08). The vN amplitude was −0.37 μV (SD = 0.99) and −1.21 μV (SD = 1.02) for the patient and control group, respectively (d = 0.83). Figure 5 graphically represents the mean amplitude and variability of these components.

**Figure 6:**
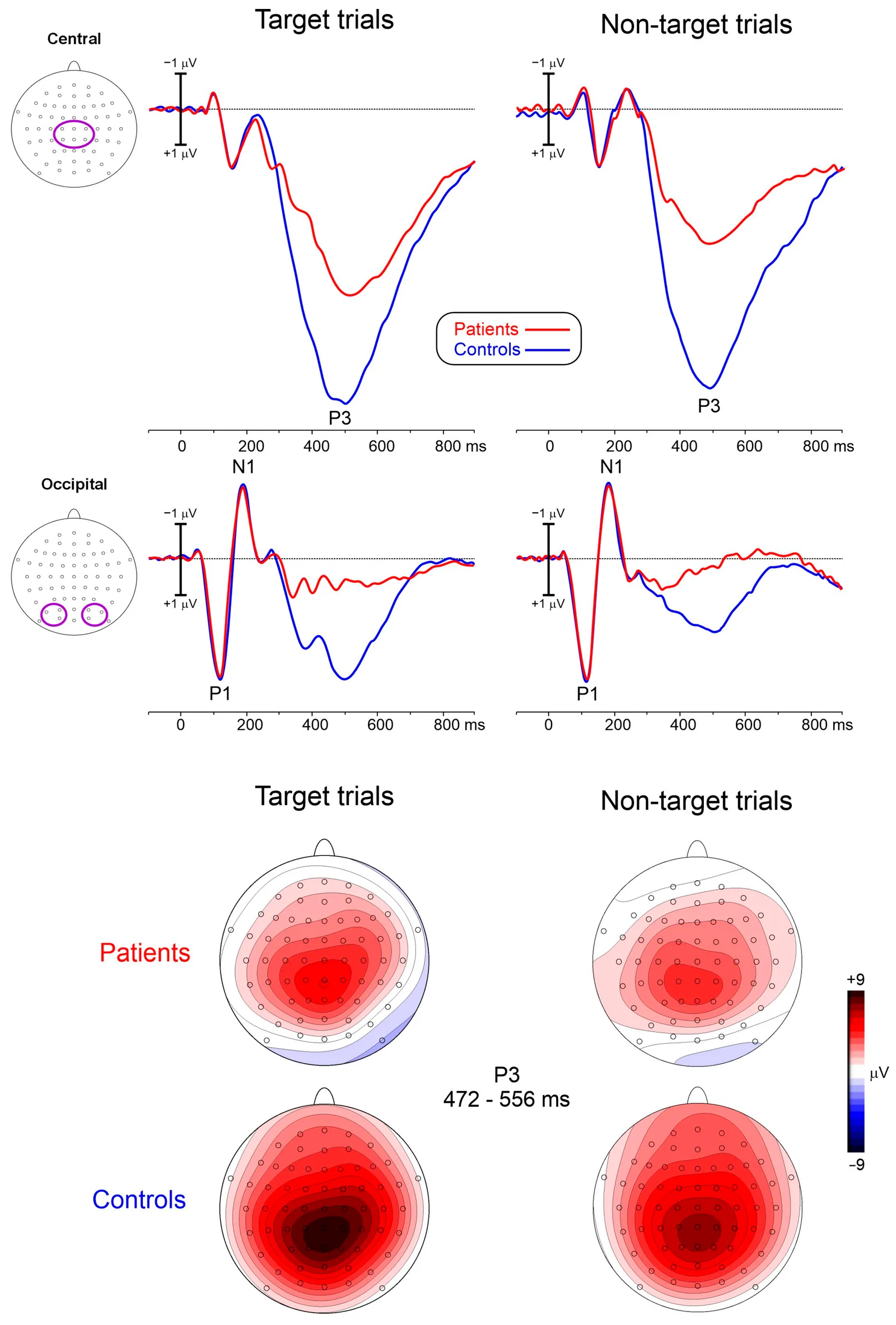
**Top**: Post-stimulus ERP waveforms of the two groups and trial types. The circles within the head representation indicate the electrodes included in the waveforms. **Below**: Voltage scalp topography (top-flat view) of the P3 in the 472–556 ms interval of the two groups and for the target and non-target trials.

Figure 6 shows the post-stimulus ERP waveforms for the two groups across the three clusters of electrodes representing the P1, N1, and P3 components. The lower panel of Figure 6 shows the voltage scalp topographies for the P3 component. Figure 6 illustrates that the absence of group differences in the P1 and N1 components was accompanied by highly similar waveform morphology and scalp topography in PTSD patients and controls, supporting the preservation of early perceptual and attentional processing stages. In contrast, the P3 component showed a marked amplitude reduction in the PTSD group across both target and non-target conditions. The scalp distributions remained comparable between groups, suggesting that the effect primarily reflected reduced activation strength rather than a change in the underlying neural configuration.

**Figure 7:**
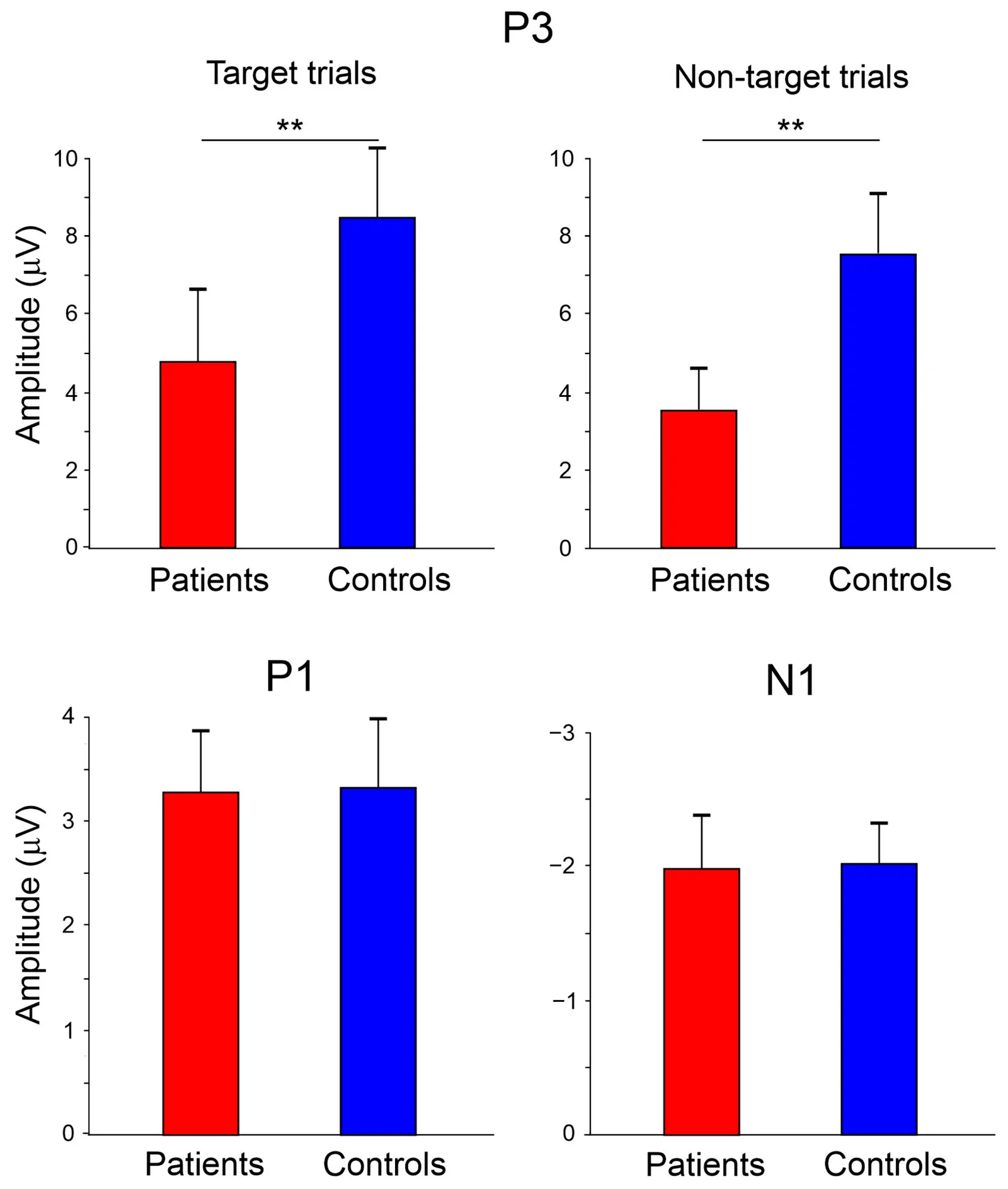
Amplitudes of the post-stimulus components and their variability expressed as the 95% confidence interval. The significant differences are also indicated (** *p* < 0.01).

The ANOVAs on the P1 and the N1 components revealed no significant effects (F < 1). However, the ANOVA on the P3 component showed a significant effect of Group (F_(1,49)_ = 10.15, *p* < 0.01, η_p_^2^ = 0.17), with lower amplitude observed in the patient group (4.15 μV, SD = 0.57) compared to the control group (7.98 μV, SD = 0.87). The effect of Trial was also significant (F_(1,49)_ = 6.24, *p* = 0.02, η_p_^2^ = 0.11), with larger amplitudes for target trials (6.60 μV, SD = 0.79) than for non-target trials (5.62 μV, SD = 0.65). The interaction between the Group and Trial factors was not significant (F < 1). Figure 7 graphically represents the mean amplitude and variability of these components.

Exploratory Pearson correlations between the considered ERP components’ amplitudes and RT/RT variability showed that RT correlated with BP and vN amplitudes, whereas RT variability correlated with pN amplitude (*p* < 0.05, R > 0.32) in both groups. No significant correlations were observed between ERP amplitudes and clinical measures, suggesting that the electrophysiological alterations were not directly related to symptom severity in the present sample.

## 4. Discussion

The present work confirms previous electrophysiological findings on PTSD patients, which suggest intact early stimulus processing and impaired post-perceptual cognitive processes during cognitive tasks using non-affective stimuli [22,23,25,29]. The present findings are also in line with more recent studies documenting persistent abnormalities of late cognitive ERP components and supporting the clinical relevance of P300-related measures as electrophysiological markers of PTSD [59,60]. This cognitive dysfunction has been associated with hypofunctional posterior parietal areas and the hippocampus, both of which are involved in the generation of the P3 ERP [61]. The P3 component is associated with post-perceptual attentional processes, such as stimulus categorization (decision-making) and task closure [62]. A reduced P3 amplitude is indicative of disturbed concentration and memory deficits [63] and has been negatively correlated with numbness symptoms [25], supporting a model that posits disordered memory and attention in PTSD [26,27,30].

The waveform and topographic patterns presented in Figure 6 further support the interpretation that PTSD-related abnormalities emerged predominantly at the stage of higher-order cognitive evaluation rather than during early sensory processing. The preserved P1 and N1 responses indicate intact perceptual encoding and attentional orienting, whereas the reduced P3 amplitude observed across target and non-target trials suggests a generalized impairment in stimulus evaluation, categorization, and task closure processes. The similarity of the scalp topographies between groups further indicates that the deficit is quantitative rather than qualitative, reflecting reduced recruitment of the neural systems contributing to P3 generation.

More importantly, the present study extends the existing literature on PTSD by investigating potential dysfunctions in anticipatory brain processing during cognitive task anticipation and motor response planning, an area that has been neglected in prior research. We examined three proactive ERP components (the BP, pN, and vN), which are associated with motor, cognitive/inhibitory, and sensory preparation, respectively, and have been localized to brain areas affected by PTSD, including the PFC, the cingulate cortex, and secondary visual areas [38].

The main finding of the study was a general suppression of these three preparatory ERP components, suggesting a broadly reduced readiness for the upcoming cognitive task. Specifically, the diminished cognitive and motor preparation (i.e., reduced BP and pN) is consistent with altered functioning in networks involving prefrontal and cingulate regions, as suggested by prior literature. The prefrontal cortex plays a crucial role in the intentional control of negative emotions, fear extinction mechanisms, and the contextualization of stimuli-processes all compromised in PTSD due to the hypo-functionality and/or volumetric reduction of these brain regions [64]. The pN component may serve as a potential electrophysiological index of prefrontal involvement in cognitive control, and its reduction may be consistent with altered functioning of prefrontal control networks [65]. Notably reduced cognitive and motor preparation has also been observed in individuals with elevated levels of anxiety [39,66], suggesting that these alterations may involve the prefrontal and cingulate cortices and could resemble those reported in PTSD.

Moreover, the inability to contextualize internal motivational states and external situational stimuli contributes to symptoms such as anxiety, rumination, avoidance, and numbness [64]. Emotional numbness, in turn, is associated with a reduced attentional response, potentially explaining why the reduced P3 amplitude was observed in PTSD [25]. The failure to interpret stimuli within the appropriate context has also been linked to dysfunction of the cingulate cortex, a region involved in voluntary action preparation and strongly interconnected with the SMA [67]. Alterations in the cingulate cortex observed in PTSD have been proposed as possible neural correlates of the disorder, while amygdala dysfunction may also reflect more general consequences of trauma exposure [68].

While these dysfunctions appear to be common to both anxious and PTSD patients, dysfunction in top-down attention during the preparatory phase of processing-evidenced by the vN component—may be particularly characteristic of PTSD. In these patients, the reduced discriminative capacity is linked to an impaired ability to process visual signals during the recognition phase, which directly impacts the ability to discern sensory features for stimuli with overlapping neural representations [69]. Visual recognition underpins the dynamic allocation of attention, and a deficit in orienting selective attention directly affects the ability to promptly discriminate stimuli and their features [70]. The vN component has a role in top-down visual attention, generating low-level mental images of the upcoming stimulus [41], and its reduction may explain difficulties in subsequent attentional processing stages.

In this study, we also investigated the behavioral consequences of proactive brain processing in a visuomotor cognitive task, specifically, response time and accuracy. Our findings suggest that cortical anomalies observed at the ERP component level seem to be reflected at the behavioral level. Reduced motor and visual preparation likely lead to longer response times [36]. Wu et al. [71] previously found prolonged response times in PTSD, interpreting them as the result of a speed-accuracy trade-off. In the present study, we confirm this trade-off, showing that the PTSD group preserved accuracy at the expense of prolonged response times. Reduced cognitive preparation in the PFC has been linked to increased response time variability [36], a pattern also observed in the current PTSD group. Importantly, increased response-time variability is not specific to PTSD, but has also been reported in ADHD, schizophrenia, major depression, and other neuropsychiatric conditions characterized by executive dysfunction and impaired top-down cognitive control [49,65,72]. The increased intraindividual coefficient of variation may be explained by impulsivity and depressive symptoms, as response inconsistency is often associated with deficits in top-down cognitive control, which may contribute to the persistence of PTSD symptoms [49,73].

Pearson correlation confirmed in both groups the association reported in the literature [36] between the RT and the ERP components BP and vN, as well as between RT variability and the pN. This may indicate that, compared to healthy people, PTSD patients show similar patterns but suppressed anticipatory activity. The absence of significant correlations between ERP measures and symptom severity suggests that the observed neurophysiological alterations may reflect a general feature of PTSD rather than the severity of specific clinical dimensions in the present sample.

In summary, PTSD is associated with reduced motor, cognitive, and visual preparation, with anxiety compromising the availability of cognitive resources necessary for task performance. Impairment in top-down cognitive control processes prevents adequate suppression of irrelevant visual representations, while prefrontal functioning may contribute to categorization difficulties, as reflected by reduced pN and P3 amplitudes. This pattern translates into symptoms of emotional numbness, leading to slower response times, but with maintained accuracy. The present work supports the hypothesis that both top-down and bottom-up attentional deficits play a critical role in PTSD, offering novel electrophysiological markers potentially related to altered prefrontal, cingulate, and visual associative network functioning in PTSD. This interpretation is consistent with recent efforts aimed at developing objective EEG- and ERP-based biomarkers for PTSD assessment, symptom characterization, and classification, particularly using P300-related neurophysiological signatures [31,59].

### 4.1. Limitations

The present study has several limitations. First, the relatively small sample size limited the possibility of conducting more detailed subgroup analyses and investigating the contribution of specific symptom clusters to the observed neurophysiological abnormalities. Second, although severe psychiatric disorders were excluded, PTSD participants exhibited varying levels of anxiety, depressive, and dissociative symptoms, making it difficult to completely disentangle PTSD-specific effects from those associated with common comorbid symptom dimensions. Third, most ERP studies in PTSD have been conducted in war veterans, whereas the present sample consisted primarily of civilians exposed to heterogeneous traumatic experiences. Although reduced P3 amplitudes appear relatively consistent across trauma types, trauma chronicity, developmental timing, and comorbid symptom profiles may influence electrophysiological findings. Therefore, caution should be exercised when directly comparing the present results with those obtained in veteran populations. Fourth, because no previous studies had investigated anticipatory ERP components such as BP, pN, and vN in PTSD, the a priori power analysis was necessarily based on previously reported P3 effects. Consequently, the study may have been optimally powered to detect differences in reactive processing rather than in the novel anticipatory measures investigated here. Finally, future research should examine the potential influence of psychotherapeutic and pharmacological treatments on both proactive and reactive electrophysiological markers and should include longitudinal designs to determine whether these ERP indices may serve as predictors of treatment response.

### 4.2. Future Directions

Future research should include larger samples to enable more refined analyses disentangling PTSD-specific effects from those of comorbid conditions such as depression, generalized anxiety, or dissociation. A dimensional approach could further clarify how individual symptom clusters (e.g., avoidance, hyperarousal, anhedonia) relate to selective impairments in proactive and reactive control. Another key direction is to examine how psychotherapeutic or pharmacological treatments modulate these neurophysiological markers, with the goal of identifying ERP indices that are sensitive to change and potentially useful as predictive or outcome biomarkers. Finally, combining ERPs with complementary methodologies, such as fMRI or EEG functional connectivity, may provide a more comprehensive understanding of the neural networks underlying cognitive dysfunction in PTSD. Furthermore, recent advances in neurofeedback interventions and EEG-based computational approaches suggest that electrophysiological markers may eventually contribute not only to diagnosis but also to treatment monitoring and prediction of clinical outcomes, supporting a precision-medicine framework for PTSD management [32,74].

## 5. Conclusions

This study provides novel electrophysiological evidence that PTSD is associated with a broad impairment of proactive cognitive control during task preparation, as shown by reduced BP, pN, and vN amplitudes. These anticipatory alterations were accompanied by preserved early perceptual and attentional processing, reduced P3 amplitude, and slower and more variable behavioral responses with preserved accuracy. Together, the findings suggest that cognitive dysfunction in PTSD involves both deficient top-down preparation before stimulus onset and impaired late-stage decision-related processing after stimulus presentation. Pre-stimulus ERP components may therefore represent promising neurophysiological markers for characterizing proactive control deficits in PTSD and for informing future longitudinal studies on treatment response and clinical outcomes.

## Data Availability

The data presented in this study are available on request from the corresponding author due to ethical restrictions and participant privacy, as the data contain identifiable neurophysiological and behavioural information subjected to institutional review board approval.
